# Honey is potentially effective in the treatment of atopic dermatitis: Clinical and mechanistic studies

**DOI:** 10.1002/iid3.153

**Published:** 2017-03-30

**Authors:** Abdullah A. Alangari, Keith Morris, Bashir A. Lwaleed, Laurie Lau, Ken Jones, Rose Cooper, Rowena Jenkins

**Affiliations:** ^1^ Department of PediatricsCollege of MedicineKing Saud UniversityRiyadhSaudi Arabia; ^2^ Department of Biomedical SciencesCardiff Metropolitan UniversityCardiffUK; ^3^ Faculty of Health SciencesUniversity of SouthamptonSouthamptonUK; ^4^ Faculty of MedicineUniversity of SouthamptonSouthamptonUK

**Keywords:** Atopic dermatitis, honey, keratinocytes, manuka, mast cells

## Abstract

**Introduction:**

As manuka honey (MH) exhibits immunoregulatory and anti‐staphylococcal activities, we aimed to investigate if it could be effective in the treatment of atopic dermatitis (AD).

**Methods:**

Adult volunteers with bilateral AD lesions were asked to apply MH on one site overnight for seven consecutive days and leave the contralateral site untreated as possible. Three Item Severity score was used to evaluate the response. Skin swabs were obtained from both sites before and after treatment to investigate the presence of staphylococci and enterotoxin production. In addition, the ability of MH and its methanolic and hexane extracts to down regulate IL4‐induced CCL26 protein release from HaCaT cells was evaluated by enzyme linked immunosorbent assay. Also, the ability of MH to modulate calcium ionophore‐induced mast cell degranulation was assessed by enzyme immunoassay.

**Results:**

In 14 patients, AD lesions significantly improved post MH treatment versus pre‐treatment as compared to control lesions. No significant changes in the skin staphylococci were observed after day 7, irrespective of honey treatment. Consistent with the clinical observation, MH significantly down regulated IL4‐induced CCL26 release from HaCaT cells in a dose‐dependent manner. This effect was partially lost, though remained significant, when methanolic and hexane extracts of MH were utilized. In addition, mast cell degranulation was significantly inhibited following treatment with MH.

**Conclusions:**

MH is potentially effective in the treatment of AD lesions based on both clinical and cellular studies through different mechanisms. This needs to be confirmed by randomized and controlled clinical trials.

## Introduction

Honey is a nutritional material that is traditionally known for its medicinal properties. It has been used in this context in diverse communities for thousands of years and is still widely popular. Recently, it has been shown that honey has broad‐spectrum antimicrobial properties both in vivo and in vitro 1, 2, 3 and has been demonstrated to promote wound healing 4. In particular, manuka honey (MH) that is mainly derived from *Leptospermum scoparium*, a shrub grown in New Zealand, was shown to interrupt cell division of *Staphylococcus aureus*, 5 the bacterium most commonly responsible for wound infections. In addition, it was shown to inhibit leukocyte infiltration, cyclooxygenase 2, and inducible nitric oxide synthase expression 6 as well as inflammation mediated through toll like receptor (TLR)1/TLR2 pathway 7. On the other hand, it may elicit pro‐inflammatory properties in the absence of active inflammation 8.

Atopic dermatitis (AD) is a common chronic atopic inflammatory skin disease characterized by intermittent episodes of intense pruritus and maculopapular rash 9. Its prevalence is 10–20% in children and 1–3% in adults and it is usually the first manifestation of a range of allergic diseases that include asthma and allergic rhinitis in a phenomenon known as the atopic march 10. Most of the immune cell types are involved in the pathogenesis of AD particularly, eosinophils, mast cells, lymphocytes, and macrophages. Keratinocytes in the epidermis also play an integral role in the pathogenesis of AD by interacting with various immune cells and stimuli from the external environment 11. For example, under the influence of IL4 from Th2 lymphocytes and macrophages, keratinocytes produce chemokine ligand (CCL) 26 (eotaxin 3), which is a major chemoattractant of eosinophils to the site of inflammation 12, 13.

*S. aureus* colonizes the skin of 70–90% of patients with AD, in contrast to only 5% of normal population 14, 15. This is due to a defect in skin barrier function, repeated scratching, and deficient cutaneous antimicrobial peptides 16. Consequently, *S. aureus* is the main cause of bacterial superinfections of AD lesions. In addition, this bacterium produces highly inflammatory exotoxins such as *α*, *β*, *γ*, and *δ* cytolysins as well as several enterotoxins (SEA to SEE) that may act as superantigens and exacerbate the on‐going inflammation 17.

The management of AD remains challenging in many patients where symptoms are not resolved by the available medications, which could also cause various adverse effects 18. Some patients prefer natural remedies and have claimed overall improvement in their symptoms when they applied honey topically on AD lesions. However, there is no clear evidence in the literature to support these claims clinically or possibly mechanistically. Because of the immunoregulatory effects of MH and its anti‐staphylococcal properties in addition to the anecdotal patients’ reports, we hypothesized that MH modulates the skin inflammation in AD.

## Methods

### Clinical study

A proof‐of‐concept, open‐label, pilot study was conducted to investigate possible effects of honey on AD lesions. Information about the study was circulated via email to all students and staff within Cardiff Metropolitan University inviting them or their adult relatives and friends with AD with bilateral similarly affected area to participate. Subjects with severe extensive AD and those with other associated skin pathology were excluded. All volunteers were provided with an outline of the study and informed consent was given prior to recruitment. Cardiff School of Health Sciences’ Research Ethics Committee at Cardiff Metropolitan University granted ethical approval.

The AD lesions were clinically scored using the Three Item Severity score (TIS), which includes erythema, edema/papulation, and excoriation. Each item is scored on a scale from 0 to 3 based on severity, so the total score could be from 0 to 9 19, 20. If the bilateral lesions were not exactly of the same severity, the slightly more severe site was chosen for honey application. Recruited volunteers were provided with a 50 g tube of Medihoney^™^ (kindly provided by Derma Sciences, UK), which is sterilized MH by γ‐irradiation; sterile gauze and a Millipore tape. On day 0 they were asked to apply a layer of honey over the treatment site at night and to cover it with gauze and remove the covering and wash the site in the morning. They were also asked to repeat this process for seven consecutive days and to leave the contralateral “control” site untreated unless their symptoms became intolerable, when they were advised to use their regular treatment including topical steroids or calcineurin inhibitors. Application of moisturizers was permitted freely on both sites. On day 7, the volunteers were re‐evaluated by taking the TIS score. Skin swabs are taken of both treated and untreated sites on days 0 and 7.

### Bacteriological studies

Each skin site involved in the study was swabbed with a Sterilin swab moistened in sterile PBS through a 2 × 2 cm square cut in a sterile acetate sheet held above the skin. The swab was immediately plated onto Mannitol Salt Agar (MSA; Oxoid, Cambridge, UK) and incubated at 37°C for 24 h. Colony color was noted, coagulase tested using Staphaurex test (Fisher Scientific Ltd, Loughborough, UK) and the identity of isolated colonies was determined with BBL^™^ crystal kits for gram positive bacteria (Becton Dickinson, Oxford, UK). Oxacillin susceptibility was determined using 5 μg discs (Oxoid) on Columbia agar plates incubated at 30°C for 24 h. Isolates were stored at −80°C on Protect beads (Technical Service Consultants Ltd, Heywood, UK) until required.

Production of A, B, C, and D enterotoxins was determined by Ridascreen R4101 (R‐biopharm, Darmstadt, Germany) according to the manufacturer's instructions. Briefly, each isolate was cultivated in 10 mL tryptone soy broth (TSB; Oxoid, Cambridge, UK) with and without a pre‐determined sub‐lethal concentration 21 of 5% (w/v) MH at 37°C for 24 h. Bacteria were removed by centrifugation at 3500*g* for 5 min at 10°C, the supernatant was filtered using 0.2 μm filter (Millipore, Watford, UK) and 100 μL was tested in the kit, which could only indicate presence or absence of the respective enterotoxin.

### Cellular studies

#### Viability assay

HaCaT cell line is regarded as a reliable model of human keratinocytes and has been used extensively to study various skin diseases. To determine the concentration of MH that will not be cytotoxic to HaCat cells, CellTiter 96^®^ AQ_ueous_ one solution cell proliferation assay was utilized (Promega, Southampton, UK) as per manufacturer's instructions. Briefly, HaCaTs were seeded in a 96‐well microplate at 10^4^ cells/well. The following day, the cells were treated with different concentrations of MH UMF 10+ (Comvita, Maidenhead, UK). The honey was filter‐sterilized using Durapore (PVDF) membrane 0.22 µm GV syringe filter (Millipore). The same honey type was used in all HaCaT experiments. The supernatant was decanted 24 h later and a 20 µL of MTS tetrazolium was added to 100 µL of PBS in each well. Spectrophotometric analysis of cell proliferation was determined at 490 nm using a plate reader (TECAN^™^, Weymouth, UK).

#### HaCaT cell line culture and stimulation

Cells were cultured using cap vented corning cell culture flasks (Sigma–Aldrich, Gillingham, UK) in Dulbecco's Modified Eagle Medium (DMEM), (Thermo Fisher Scientific, Loughborough, UK) supplemented with 10% fetal calf serum (FCS) v/v, 100 IU penicillin and 0.1 mg/mL streptomycin mix (1%, v/v), and 2 mM glutamine (1%, v/v) (Sigma–Aldrich). At about 80% confluence, the cells were detached with trypsin and seeded in 12‐well plates at 0.5 × 10^6^ cells/mL/well until they achieved 70–80% confluence. The cells were then treated with 1% (w/v) MH. IL4 (Thermo Fisher Scientific) 50 ng/mL was applied 2 h later.

#### Cytokines measurement

At 24‐h post‐treatment with IL4 the supernatant was retrieved. CCL26 and IL8 concentrations were measured using Quantikine ELISA system (R&D Systems, Abingdon, UK) as per manufacturer's instructions.

#### RT‐PCR

RNA was extracted from HaCaT cells using Trizol (Invitrogen, Loughborough, UK). RNA quantity and quality was estimated using Nanodrop^™^ spectrophotometer. mRNA was converted into cDNA using high capacity cDNA reverse transcription kit (Applied Biosystems, Loughborough, UK) and 4.5 µL of cDNA was used in each 10 µL PCR reaction. Taqman PCR method was used with denaturation at 95°C for 5 min, followed by 34 cycles of denaturation at 95°C for 1 min, annealing at 65°C for 1 min, extension at 72°C for 1 min, and a final elongation at 72°C for 10 min. GAPDH was used as housekeeping gene. Forward and reverse primers for CCL26, IL8, and GAPDH were also obtained from Applied Biosystems. Relative gene expression was determined using the standard ΔΔCt method.

#### Western blotting

Signal Transducer and Activator of Transcription 6 (STAT6) phosphorylation was estimated by Western Blotting. Briefly, HaCaT cells were cultured in 8‐well plates at 1 × 10^6^ cells/well then next day were treated with or without honey and 2 h later treated with IL4. They were lysed 1 h post‐treatment with IL4 using RIPA buffer with protease and phosphatase inhibitors cocktails (all from Thermo Fisher Scientific). The cell lysate was kept in ice for 30 min then sonicated, centrifuged and stored at −80°C for later use. Total protein was estimated in the samples using protein assay kit (Biorad, Watford, UK) according to manufacturer's recommendation. After sample preparation, NuPAGE 10% bis‐tris gels (Invitrogen) were loaded with 40 µg of protein per lane and run with 165 volts constant voltage. The protein was transferred to nitrocellulose membrane using iBlot system (Invitrogen). The membrane was incubated with antibodies (Cell Signaling, Hitchin, UK) to p‐STAT6 (1:1000), STAT6 (1:1000), then β‐actin (1:2000) dilutions. The membrane was stripped after each development with Restore Plus stripping buffer (Thermo Fisher Scientific). Goat F(ab')_2_ anti‐rabbit IgG F(ab')_2_ was used as secondary antibody in p‐STAT6 blot at 1:2000, STAT6 at 1:10,000, and beta actin at 1:20,000 dilutions. Amersham ECL prime was used as detection reagent and the blot was developed on Amersham Hyperfilm ECL (Healthcare Life Sciences, Amersham, UK).

#### Methanol and hexane extracts preparation

Methanol or hexane were mixed with MH separately (1:1 w/v) and homogenized by vortexing. The mixture was centrifuged at 3000*g* for 15 min and the supernatant was aspirated and blown to dryness under N_2_. The methanol extract was in liquid form and the hexane extract was in solid form that was dissolved in 10 µL DMSO (Sigma–Aldrich) per 1 g of honey. Extracts were adjusted to original volume of solvent used by adding DMEM and that was considered 100%.

#### Mast cell degranulation assay

LAD‐2 human mast cell line was used to study mast cell degranulation in vitro and its inhibition by Medihoney^™^. Cells were cultured in a serum‐free medium (StemPro‐34 SFM, Invitrogen), which was supplied complete with L‐glutamine, penicillin, streptomycin, and stem cell factor. They were pre‐treated with 0.5%, 1%, and 2% Medihoney^™^ for 20 min at 37°C, 5% CO_2_ in air then challenged with calcium ionophore‐A23187. The concentration of stimulated histamine release was determined using an enzyme immunoassay according to the manufacturer's instructions (Beckman Coulter, High Wycombe, UK).

### Statistical analysis

Paired Student's *t*‐test was used to compare groups in the clinical study. For experimental studies, one or two‐way ANOVA with Turkey's multiple comparison tests or Student's *t*‐test were applied. Statistical analysis was undertaken using GraphPad Prism 6.0.

## Results

### Clinical study

**Figure 1:**
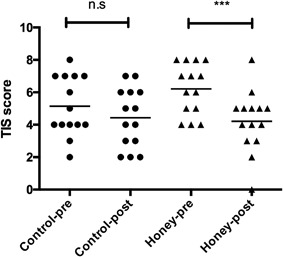
Three Item Severity (TIS) score of MH treated and control sites pre‐ and post‐treatment. The mean TIS for sites treated with MH was significantly lower than that prior to treatment. n.s, not significant.

Twenty‐six individuals volunteered for the study. After screening, 10 individuals did not have AD or did not have two similarly affected areas and were excluded. Sixteen participants were recruited and two withdrew one to two days following recruitment due to worsening symptoms. Fourteen patients completed the study. Their mean age (±SD) was 33 ± 10 years, eight were females. There was no difference at baseline in the mean TIS score between treatment and control sites. The mean TIS score of honey treated lesions was significantly less post‐treatment as compared to pre‐treatment with mean difference = −2 points, 95%CI (−2.75, −1.25), *p* <0.001 (Fig. 1), whereas there was no significant difference in control lesions between pre‐ and post‐treatment scores with mean difference = −0.7 points, 95%CI (−1.71, 0.28), *p* = 0.15. Only two patients reported using topical steroid on the control site because of intolerable symptoms. Interestingly, a one year follow up of volunteers by phone calls revealed that three of them (numbers 2, 8, and 13) reported overall improvement of their eczema without using MH after the study period.

### Bacteriological studies

Staphylococci were isolated from 25 (89%) of the 28 volunteer sites sampled on recruitment to the study (day 0) and from 21/28 (75%) sites on day 7 (Table 1). Honey treatment did not change the skin bacterial flora. Enterotoxin production in vitro was detected in only six isolates, which were cultures of *S. aureus* isolated from participants 4, 6, 12, and 14 (Table 1). Enterotoxins B, C, and D were below detectable levels when *S. aurues* isolated from the treated sites of volunteers 12, 6, and 4, respectively, were each cultivated with Medihoney^™^. However, enterotoxin C and D were unaffected in the strains recovered from the untreated sites of participant 6 and 14, respectively. Levels of enterotoxins A and E were not abolished by honey treatment in vitro in any tested strain.

**Table 1:** Effect of honey treatment on cultured staphylococci from the patients’ skin

Volunteer number	Site tested	Staphylococci recovered on Day 0	Staphylococci recovered on Day 7	Enterotoxin detected without honey*	Enterotoxin detected in the presence of 5% (w/v) honey*
2	Untreated	*S. haemolyticus*	*S. haemolyticus*		
2	Treated	*S. haemolyticus*	*S. saprophyticus*		
3	Untreated	*S. haemolyticus*	*S. haemolyticus*		
3	Treated	*S. haemolyticus*	*S. epidermidis*		
4	Untreated	*S. aureus*		A, E	A, E
4	Treated	*S. aureus*	*S. aureus*	A, D, E	A, E
5	Untreated		*S. aureus*		
5	Treated	*S. aureus*	*S. aureus*		
6	Untreated		*S. aureus*	C	C
6	Treated	*S. aureus*	*S. aureus*	C	
7	Untreated	*S. simulans*			
7	Treated	*S. haemolyticus*			
8	Untreated	*S. haemolyticus*	*S. haemolyticus*		
		*S. epidermidis*			
8	Treated	*S.saprophyticus*			
		*S. intermedius*			
9	Untreated	*S. aureus*	*S. aureus*		
9	Treated	*S. aureus*	*S. haemolyticus*		
10	Untreated	*S. kloosii*			
10	Treated			No isolate to test	No isolate to test
12	Untreated	*S. aureus*	*S. aureus*		
12	Treated	*S. aureus (MRSA)*	*S. aureus*	B	
13	Untreated	*S. aureus*	*S. aureus*		
13	Treated	*S. aureus*	*S. aureus*		
14	Untreated	*S. haemolyticus*	*S. aureus*		
			*S. capitis*		
14	Treated	*S. aureus*	*S. aureus*	A, D, E	A, D, E
		*S. haemolyticus*	*S. auricularis*		
15	Untreated	*S. aureus*	*S. aureus*		
15	Treated	*S. aureus*	*S. aureus*		
		*S. capitis*			
16	Untreated	*S. aureus*	*S. aureus*		
		*S. haemolyticus*	*S. capitis*		
16	Treated	*S. aureus*	*S. aureus*		
			*S. capitis*		

* Honey treatment in vitro of the isolated *S. aureus*.

### Cellular studies

#### Viability of HaCaTs treated with different concentrations of MH

**Figure 2:**
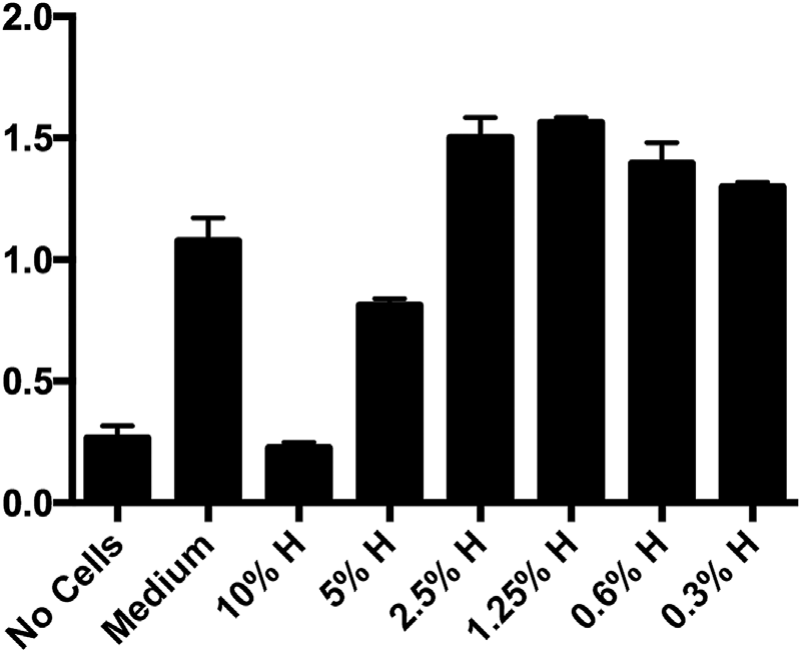
HaCaT cells viability after treatment with different concentrations of honey (w/v) using MTS assay. Y‐axis indicates absorbance reading. Bars represent the Mean ± SD. (*n* = 3). H, honey.

After 24 h of MH treatment, the maximum concentration of honey tested that did not reduce HaCaT viability was 2.5% (w/v; Fig. 2). There was, in fact, increased proliferation at this concentration, which could be attributed to the sugar content of honey. We chose in most of the following experiments to work with 1% honey (w/v) to be consistent with previous studies.

#### Effect of MH treatment on IL4‐induced CCL26 secretion

**Figure 3:**
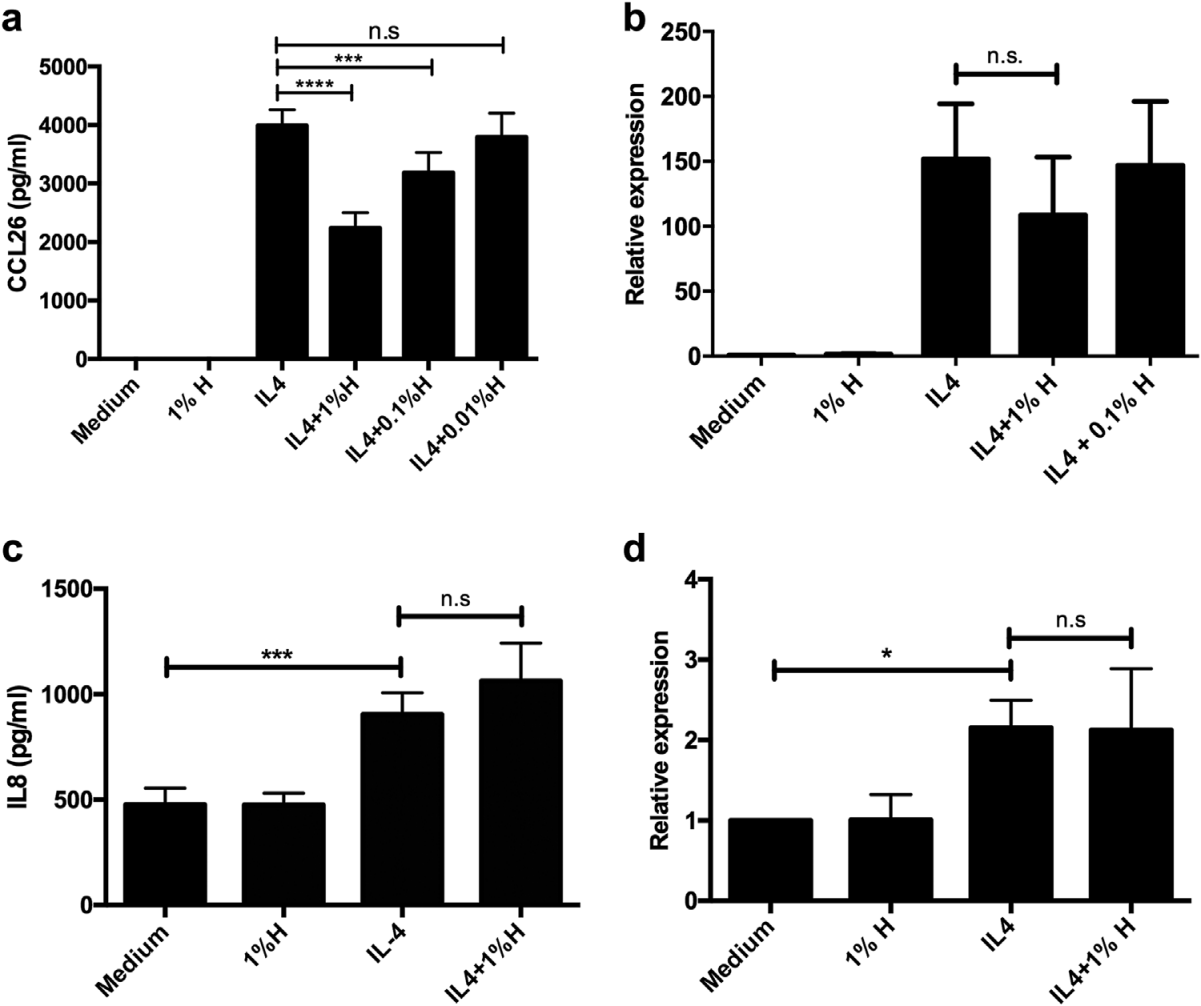
Effect of 1% honey pre‐treatment of HaCaTs on (a) IL4‐induced CCL26 protein release, and (b) m‐RNA expression (GAPDH was used as house keeping gene); (c) IL4‐induced IL8 protein release, and (d) m‐RNA expression (GAPDH was used as house keeping gene). Bars represent the Mean ± SD. (*n* = 3). H, honey; n.s, not significant.

To investigate whether MH could down regulate IL4‐induced CCL26 secretion by HaCat cells, HaCaTs were treated with IL4 50 ng/mL 2 h after the application of 1%, 0.1%, or 0.01% honey. We found that honey could significantly down regulate CCL26 secretion in a dose‐dependent manner (*p* < 0.001; Fig. 3a).

#### CCL26 mRNA expression

IL4 up regulates CCL26 secretion by inducing its m‐RNA transcription. To investigate whether MH effect on CCL26 secretion is mediated through interruption of this process, we performed RT‐PCR. There was a trend of reduction of IL4 induced CCL26 m‐RNA expression after pre‐treatment with 1% honey as compared to no treatment or 0.1% honey treatment. This, however, was not significant (*p* = 0.29, Fig. 3b). GAPDH was used as house keeping gene. Similar results were obtained with GUSB as house keeping gene (data not shown).

#### Effect of MH treatment on IL4‐induced IL8 secretion

Because of the diverse contents of MH, the generalizability of the above effects on multiple cytokines was investigated. IL8 protein levels in the supernatant fluid as well as m‐RNA expression from the samples of the above experiment were assessed. IL4 significantly induced IL8 release (*p* < 0.001) and m‐RNA expression (*p* = 0.03). However, we could not observe any effect of honey on IL4‐induced IL8 at either the protein or m‐RNA levels (Fig. 3c,d).

#### Effect of MH treatment on IL4‐induced STAT6 phosphorylation

**Figure 4:**
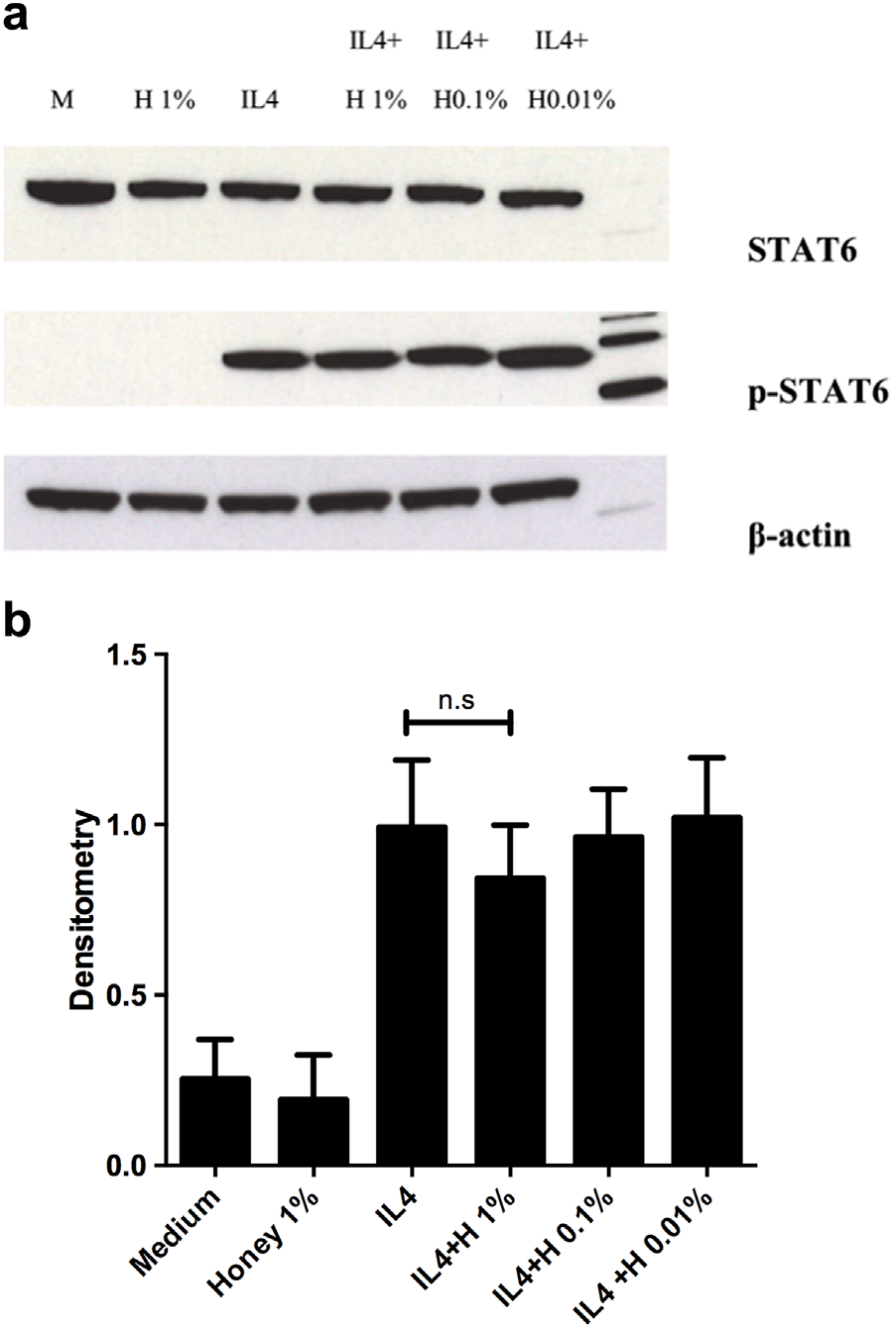
(a) Western Blotting showing no visible effect of pretreatment of HaCaTs with 1% honey on IL4‐induced STAT6 phosphorylation. (b) Densitometry analysis of p‐STAT6/STAT6 ratio. Bars represent the Mean ± SD. (*n* = 3). M, medium; H, honey; n.s, not significant.

Since the only known pathway of CCL26 induction by IL4 involves phosphorylation of STAT6 transcription factor 22, 23, the ability of MH to down regulate STAT6 phosphorylation was investigated. HaCaTs were treated with IL4 (50 ng/mL) with or without pretreatment with incremental concentrations of honey 2 h earlier (Fig. 4a,b). Analysis of the p‐STAT6/STAT6 densitometry ratio did not reveal any significant down regulation of IL4‐induced STAT6 phosphorylation following treatment with MH (*p* = 0.78), although there was a small trend of decrement with 1% honey treatment.

#### Effects of methanol and hexane honey extracts on IL4‐induced CCL26

**Figure 5:**
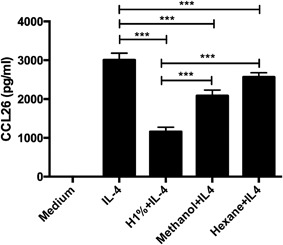
Effect of pre‐treatment with 1% honey, 1% honey methanol extract, and 1% honey hexane extract on IL4‐induced CCL26 release from HaCaTs. Bars represent the Mean ± SD. (*n* = 3). H, honey.

To investigate which components of MH caused its effect on CCL26 release from HaCaTs, we prepared methanol and hexane extracts of whole MH. Methanol is a very polar solvent with polarity index of 5.1% and 100% water solubility, while hexane is a nonpolar solvent with polarity index of 0.1% and 0.001% water solubility. Both 1% (w/v) methanol and 1% (w/v) hexane extracts were able to significantly down regulate IL4‐induced CCL26 release from HaCaTs, but significantly less than whole honey (Fig. 5).

#### Effect of honey treatment on mast cells degranulation

**Figure 6:**
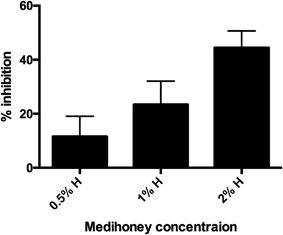
Histamine inhibition assay showing dose‐dependent inhibition of LAD‐2 mast cell histamine release by Medihoney^™^. Bars represent the Mean ± SD. (*n* = 3). H, honey.

Pre‐treatment of LAD2 cells with Medihoney^™^ lead to a dose‐dependent inhibition of histamine release following calcium ionophore‐A23187 stimulation (Fig. 6).

## Discussion

We herein report a number of novel findings: a pilot clinical study suggesting the efficacy of MH in the treatment of AD as well as mechanistic data that support this clinical finding including down regulation of IL4‐induced CCL26 release from keratinocytes and inhibition of mast cells degranulation. We have also shown that the effect of honey is likely to be mediated by multiple components of different physical and chemical properties.

CCL26 plays a key role in the pathogenesis and severity of AD 24, 25 as well as other conditions were eosinophils are a major contributor such as asthma 26 and eosinophilic esophagitis 27. It is more potent than eotaxin1 (CCL11) and eotaxin2 (CCL24) in attracting eosinophils 28. Chemokine receptor (CCR)3 is a common receptor to all three chemokines 29, its expression is up regulated in AD lesions 30, and its blockade by monoclonal antibodies inhibits eosinophils recruitment 31. CCR3 is also expressed on basophils 32, mast cells 33, and activated Th2 cells 34. Therefore, honey's significant down regulation of IL4‐induced CCL26 release by keratinocytes could explain, at least partly, our clinical findings. However, we could not show significant inhibition of STAT6 phosphorylation nor could we able to show significant down regulation of CCL26 mRNA expression by MH following IL4 stimulation of HaCaTs. This suggests that MH may exhibit its effects primarily at CCL26 translational or post‐translational levels, and requires further study. Moreover, as methanolic and, to a lesser extent, hexane extracts of MH retain some of the activity of whole MH; it is likely that the effect of whole MH is the sum of several of its multiple constituents. These components possibly include polyphenolic compounds 35 that we have found to exhibit anti‐inflammatory effects within MH methanolic extract (Wythecome et al., manuscript under revision) and other lipid‐soluble compounds yet to be defined. For example, thiazolidinediones such as troglitazone and rosiglitazone, which are peroxisome proliferator‐activated receptor *γ* (PPARγ) agonists and structurally similar to some flavonoids found within MH, have been shown to reduce IL4‐induced eotaxin release dose dependently, though via an unknown mechanism 36.

IL4 can also induce IL8 release from epithelial cells 37, 38. This effect is possibly mediated through p38 mitogen‐activated protein kinase (MAPK), extracellular signal‐regulated kinase (ERK) pathway 39, rather than STAT6 pathway 40. MH did not modulate IL4‐induced IL8 release at the protein or mRNA levels. These findings suggest that even with the large diversity of MH components, its overall effects seem to be targeted toward certain pathways.

Mast cells are found in increasing numbers in the epidermis and dermis of patients with AD 41. They contribute to the signs and symptoms of the disease through the release of mediators such as histamine from granules causing itching, local redness, and edema 42 as well as disturbing skin barrier integrity 43. The dose‐dependent inhibition of histamine release by MH could partly explain its clinical effects. However, its mechanism remains to be elucidated.

Treatment with MH did not alter the skin culture results of *Staphylococci*, but we observed in vitro inhibition of some enterotoxins release namely, SEB, SEC, and SED from cultured *S. aureus* that were obtained from three different subjects. However, this inhibition did not correlate with the clinical improvement in those particular subjects and was not consistent in cases of SEC and SED. Nevertheless, this needs further study. Recently it has been shown in methicillin‐resistant *S. aurues* (MRSA) that virulence genes were down‐regulated in vitro following exposure to MH, with the greatest effect on *sec3*, a gene that codes for SEC 44.

Our studies have some limitations. The patient number in the clinical study is small and the scoring system applied, although validated and correlates with more complex and widely used scores 19, 20, is not the gold standard of AD clinical assessment, but is useful in evaluating specific lesions rather than global response. It could also be argued that not covering the control site may have had influenced the outcome through patients’ scratching. However, the treated site was only covered overnight. In addition, participants had the liberty to treat the control site with their conventional treatment at any time if they feel their symptoms were intolerable and covering the control site would have prevented them from doing so. Moreover, the cover of the honey treated site was light and would not have prevented participants from itching. In addition, the mechanism by which MH inhibits IL4‐induced CCL26 release form keratinocytes is still unclear. It was not also possible to decipher the active ingredients in MH related to our findings.

In conclusion, honey is a very complex material with potential therapeutic value in the treatment of AD. Future research should aim to investigate whether similar effects can be reproduced with other honey types. Producing more practical form of honey to use topically on the skin should hasten clinical investigations. In addition, these findings should open the door to the potential role of honey in the treatment of other atopic conditions like asthma or allergic rhinitis.

## Conflict of Interest

R. Cooper received consultation fees, sponsorship to attend scientific meetings, and remuneration for presentations from Derma Sciences Inc. and Comvita, UK. Other authors have nothing to disclose in relation to this manuscript.
